# EXPLORING DEATH AND DYING WITH HEALTHCARE STUDENTS

**DOI:** 10.1093/geroni/igz038.2662

**Published:** 2019-11-08

**Authors:** Megan Foti, Stacy Cassel

**Affiliations:** Stockton University, Galloway, New Jersey, United States

## Abstract

Although death is a universal experience, many avoid discussing or learning more about the topic (Mak, 2011). However, healthcare professionals are expected to be knowledgeable, resourceful, and professional within their scopes of practice, but oftentimes avoid end of life topics (Ramvi & Gripsrud, 2017). Students also report concerns when working with clients at the end of life expressing fear of how they will handle an encounter with a dying client (Ek et al., 2014). Therefore, professionals need didactic and personal preparation to be more comfortable with discussions related to end of life treatment and planning (Kumar et al, 2013). Evidence supports that students can benefit from self-reflective and narrative exercise with older adults that challenge their perspectives on end of life. Students who are able to openly discuss death, dying, illness and loss express a desire to learn more about working with the older adult population and increased sensitivity to the beliefs and attitudes of older adults (Butler & Baghi, 2008; Nelson et al., 2018). In order to provide students with an opportunity to explore end of life topics, professors designed an educational module which included activities such as writing a living will, discussing end of life topics with older adults, and critically reflecting on their experiences. This proposed poster will highlight current background literature relevant to end of life topics, methods for integrating end of life topics into academic curricula, and student perceptions related to end of life topics as shared in their reflections.

